# Tumor Necrosis Factor-Alpha and Interleukin 6 in Human Periapical Lesions

**DOI:** 10.1155/2007/38210

**Published:** 2006-12-27

**Authors:** Ivana Brekalo Pršo, Willy Kocjan, Hrvoje Šimić, Gordana Brumini, Sonja Pezelj-Ribarić, Josipa Borčić, Silvio Ferreri, Ivana Miletić Karlović

**Affiliations:** ^1^Department of Dentistry, Medical Faculty, University of Rijeka, HR 51000 Rijeka, Croatia; ^2^Department of Histology, Medical Faculty, University of Rijeka, HR 51000 Rijeka, Croatia; ^3^Department of Informatics, Medical Faculty, University of Rijeka, HR 51000 Rijeka, Croatia; ^4^School of Dental Medicine, University of Zagreb, HR 10000 Zagreb, Croatia

## Abstract

*Aim*. The aim of this study was to evaluate the presence of the cytokines tumor necrosis factor-alpha (TNF-*α*) and interleukin-6 (IL-6) in human periapical lesions. *Subjects and methods*. Samples were obtained from three groups of teeth: symptomatic teeth, asymptomatic lesions, and uninflamed periradicular tissues as a control. *Results*. TNF-alpha levels were significantly increased in symptomatic lesions compared to control. Group with asymptomatic lesions had significantly higher concentrations compared to control. There were no significant differences in TNF-alpha levels between symptomatic and asymptomatic lesions. In group with symptomatic lesions, IL-6 levels were significantly higher than in group with asymptomatic lesions. The IL-6 levels in symptomatic group also showed significantly higher concentration in comparison with control group. In asymptomatic group, the IL-6 level had significantly higher concentrations compared to control. *Conclusion*. These results indicate that symptomatic lesions represent an immunologically active stage of disease, and asymptomatic lesions are the point from which the process advances toward healing.

## 1. INTRODUCTION

Periapical inflammatory lesions are a frequent pathology and, in most cases, a consequence of dental caries. This type of lesion develops as an immune reaction triggered by the presence of bacteria in the root canal and bacterial toxins in the periapical region [1]. After microbial invasion of periapical tissues, both nonspecific and specific immunologic responses persist in the host tissues. This inflammatory process ultimately results in destruction of the alveolar bone surrounding the tooth. It is characterized by the presence of immunocompetent cells producing a wide variety of inflammatory mediators. TNF-alpha is a soluble mediator and is released from immunocompetent cells in inflammatory processes. TNF-alpha plays an important role in initiating and coordinating the cellular events that make up the immune system's response to infection. The biological effects of TNF-alpha include activation of leukocytes such as lymphocytes (T and B cells), macrophages, and natural killer cells; fever induction; acute-phase protein release; cytokine and chemokine gene expression; and endothelial cell activation [2]. IL-6 is a pleotropic cytokine that influences the antigen-specific immune responses and inflammatory reactions. It stimulates the formation of osteoclast precursors from colony-forming unit-granulocyte-macrophage and increases number of osteoclasts in vivo, leading to systemic increase in bone resorption. Emerging data suggests that IL-6 also has significant anti-inflammatory activities [3]. Together with IL-1 and TNF*α* (which also stimulate IL-6 secretion), it belongs to the group of main proinflammatory cytokines [4].

The inflammatory response in the persisting apical lesion protects the host from further microbial invasion. The pathogenic pathways linking infection with development of a periapical lesion and concomitant bone resorption are not fully understood. Large numbers of immunocompetent cells such as macrophages, activated T and B cells and plasma cells synthesizing all classes of immunoglobulins are present in periapical lesions [5].

The various activities of IL-6 and TNF-alpha suggest that these factors could play a major role in mediation of the inflammatory and immune responses initiated by infection or injury [6]. The aim of this study was to determine the TNF-alpha and IL-6 levels in symptomatic and asymptomatic periapical lesions using an enzyme-linked immunosorbent assay (ELISA) in surgically removed human periapical lesions.

## 2. SUBJECTS AND METHODS

A total number of 45 teeth were included in this study. The teeth were divided into three groups. Group 1 consisted of lesions from 15 teeth that had been diagnosed as symptomatic. Teeth were put in this group based on the following criteria: clinical and radiographic examination that determined the existence of periradicular pathosis involving destruction of cortical bone and painful sensitivity to percussion and/or palpation [5].

Group 2 consisted of lesions from 15 teeth that had been diagnosed as asymptomatic. A diagnosis was made based on the following criteria: clinical and radiographic examination that determined the existence of periradicular pathosis involving destruction of cortical bone, no or slight sensitivity to percussion.

Group 3 consisted of uninflamed periradicular tissues that were obtained from periapical regions of 15 unerupted and incompletely formed third molars. The teeth included in this group had to meet the following criteria: a verbal history confirming no history of pulpal pain, clinical and radiographic examination after extraction assuring that these teeth had no caries.

Clinical examination was performed according to the standard clinical criteria. After informed consent had been obtained and medical, dental, and social histories collected, tissues were obtained by apicoectomy. The diameter of the lesions, determined on the radiographs, ranged from 2 mm to 16 mm.

The surgery was performed with the patients under local anesthesia. The patients involved in this study had not suffered from any diseases requiring any form of medical treatment except for dental surgery. These patients did not receive any medications including salicylates, nonsteroid anti-inflammatory drugs, or antibiotics for about 1 month prior to surgery. After excision of the lesion, each specimen was divided into two. One section was taken for histopathological evaluation and was stained with hematoxylin and eosin. Histological examination showed that 25 tissue samples were granulomas comprising of connective tissue with variable collagen density, inflammatory infiltrate predominantly of macrophages, lymphocytes, and groups of plasmocytes, polymorphonucleocytes and giant cells, as well as the presence of fibroangioblastic proliferation in variable degrees. Three lesions were diagnosed as scar tissue. Two lesions presented connective tissue with variable diffuse inflammatory infiltrate and cavity formation limited by continuous or discontinuous stratified squamous epithelium, and thus were considered inflammatory cysts. In the samples of symptomatic lesions, there was a presence of polymorphonuclear cells, more than in asymptomatic lesions.

Before homogenization every sample was weighed. For cytokine analysis, the tissue was cut up finely with scissors and homogenized in a glass tissue grinder with a Teflon plunge. The elutions were performed at 4°C over a 30 minute period with mixing before centrifugation for 2 minutes at 9880 g. The concentrations of TNF-alpha and IL-6 were analyzed with a commercial enzyme-linked immunosorbent assay kit (ELISA; R&D, Minneapolis, Minn, USA). The assay was performed according to the manufacturer's instructions and the results are expressed in pg/mL. The detection limit for TNF-alpha was 4.4 pg/mL and 1.4 pg/mL for IL-2, respectively. Results of the protein content were expressed in log 10 pg/mL.

All subjects were informed of the aims and procedures of research, as well as of the fact that their medical data would be used in research. Within the research they were guaranteed respect of their basic ethical and bioethical principles—personal integrity (independence, righteousness, well-being, and safety) as regulated by Nűrnberg codex and the most recent version of Helsinki declaration. Only those subjects who have given a written permission in form of informed consent were included.

## 3. STATISTICAL ANALYSIS

Data are presented as median values, interquartile range (IQR) and on a logarithmic scale. The results obtained were compared using the nonparametric Kruskal-Wallis test and Mann-Whitney as a post-hoc test.

All statistical values were considered significant at the *P* level of .05. Statistical analysis of data was performed by using Statistica for Windows, release 6.1 (StaSoft Inc., Tulsa, Okla, USA).

## 4. RESULTS

This study quantified the levels of TNF-alpha and IL-6 in symptomatic and asymptomatic human periapical lesions. Lesions were also categorized by the size and histological findings.

**Figure 1:**
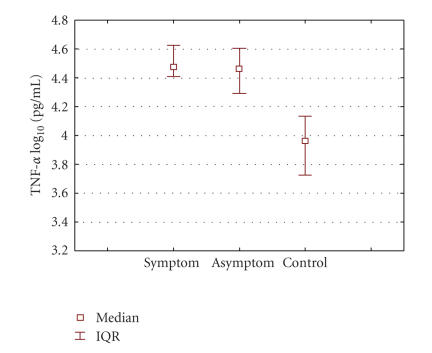
Graphic representation of median TNF-alpha concentrations in symptomatic, asymptomatic, and control groups. Data represent the median log 10 cytokine contents (IQR) per milliliter. Significance: symptomatic group compared to control: median 4.47 (IQR: 4.44–4.61) pg/mL versus 3.96 (IQR: 3.86–4.10) pg/mL; *P* < .001. Group with asymptomatic lesions compared to control: median 4.46 (IQR: 4.28–4.60) pg/mL versus 3.96 (IQR: 3.86–4.10) pg/mL; *P* < .001.

**Figure 2:**
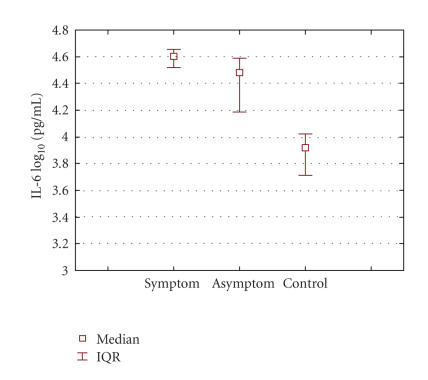
Graphic representation of median IL-6 concentrations in periapical lesions according to clinical diagnosis. Data represent the median log 10 cytokine contents (IQR) per mililliter. Significance: symptomatic and asymptomatic groups: median 4.60 pg/mL versus 4.48 pg/mL; *P* = .002. Symptomatic group and control group: median 4.60 pg/mL versus 3.91 pg/mL; *P* < .001. Asymptomatic group compared to control: median 4.48 pg/mL versus 3.91 pg/mL; *P* < .001.

The levels of TNF-alpha and IL-6 were measured in the symptomatic and asymptomatic human periapical lesions as well as in the control group, and are presented as the median (IQR) on a logarithmic scale in Figures 1 and 2. Median value for TNF-alpha in the symptomatic group: 4.47 (IQR: 4.44–4.61) pg/mL was significantly higher (*P* < .001) compared to 3.96 (IQR: 3.86–4.10) pg/mL in control group. Median value of TNF-alpha in the asymptomatic group: 4.46 (IQR: 4.28–4.60) pg/m was also significantly higher (*P* < .001), compared to 3.96 (IQR: 3.86–4.10) pg/mL in control group. There was no significant difference in TNF-alpha level between symptomatic and asymptomatic lesions: median 4.48 pg/mL versus 3.91 pg/mL (*P* = .418). (Figure 1).

## 5. DISCUSSION

Periapical lesions usually result from a persistent inflammatory response induced by prolonged exposure of periapical tissues to various microbial agents, evoking an immunological reaction. In this local defense mechanism, various inflammatory mediators, in particular inflammatory cytokines IL-6 and TNF-alpha, play a complex and central role in regulation of the immune response.

The immune complex is formed by cells whose main function is to recognize antigens that penetrate the organism and to neutralize and/or destroy them [7]. IL-6 has many molecular forms and each molecule has a different function when secreted by different cells in distinct situations (activated through diverse stimuli). Several studies have shown that both humoral and cellular immune responses play important roles in the pathogenesis of periapical lesions [8]. The cytokine expression has been investigated in periapical lesions, however, the role that these molecules may play in the pathogenesis of the disease has not been well established.

The inflammatory cytokines IL-6 and TNF-alpha have been demonstrated to have the capacity to activate osteoclastic bone resorption [9].

The mediators involved in the inflammatory process and bone resorption appear to be more complex. Thus, human and animal studies have demonstrated the active participation of other cytokines, such as TNF-alpha, IL-6, IL-3, GM-CSF, IL-11, IL-17, and IL-18, which have shown their potential role in the pathogenesis of osteolytic diseases [9, 10]. These cytokines might be acting synergistically with IL-1, promoting activation/differentiation of osteoclasts and production/secretion of prostaglandins by many cell types, including fibroblasts and osteoblasts [10]. IL-6 has traditionally been considered to be a proinflammatory mediator, since it is induced by IL-1 and TNF-alpha early in the inflammatory cascade, and because it stimulates expression of acute-phase proteins [3].

Our results demonstrate the presence of IL-6 in the vast majority of tissue samples and are in agreement with those from previous studies [11]. It has been reported that cystic growth may be due to the autocrine stimulation of cyst epithelial cell proliferation by TNF-alpha and IL-6, and the osteolytic activity of these cytokines, causing local bone loss [6]. In this study, the concentrations of IL-6 in the symptomatic lesions were statistically significantly in correlation with asymptomatic lesions and control group. These results suggest that these lesions may represent an active state of inflammatory periradicular disease which has already been confirmed [11, 12]. The plasma concentration of IL-6 has also been reported to correlate with severity of infection in certain clinical pathologic conditions [12]. The levels of IL-6 have also been measured in the patients with atypical painful disorders in orofacial region, and their levels were significantly greater than those in control group [13].

Results from the previous studies confirm the results obtained in this study, since it has been proved at different levels that IL-6 is significantly increased in certain infections and painful conditions. In researches performed to the present date, it has been proved that both IL-6 and TNF-alpha are produced in response to infectious organisms, in vitro and in vivo conditions. Once produced, they could exert a beneficial or deleterious effect, depending on the quantity in which they are produced and the time period over which production is sustained [3].

In our study, TNF-alpha and IL-6 were detected in all of the periapical samples. Highest concentration of TNF-alpha was detected in symptomatic and asymptomatic lesions, while lowest TNF-alpha concentration was found in healthy samples. Somewhat greater concentration was found in symptomatic lesions, but there was no statistically significant difference between symptomatic and asymptomatic groups. Statistically significant difference was found in symptomatic and asymptomatic groups in comparison with the control group shows clearly that TNF-alpha is an important bone-resorptive mediator and its elevated levels have far-reaching systemic consequences. Inflammatory cytokines also play a part in the modulation of pain by interfering with nociceptive transduction, conduction, and transmission. This modulation may result from alteration of the transcription rate and post-translational changes in proteins involved in the pain pathway. In the previous study, an important role was assigned to IL-6 in the physiology of nociception and the pathophysiology of pain [14]. Because of this fact, we wanted to analyze the correlation between tissue cytokine levels and characteristic features of the lesions, such as symptoms. Samples in Group 1 represent a symptomatic group. The elevated IL-6 and TNF-alpha levels in this group suggest that these lesions could represent an active state of inflammatory periradicular disease. In Group 2 which is the asymptomatic group, we found significantly elevated levels of both cytokines, compared to the control group. However, the difference in IL-6 level between this group and both symptomatic and control groups was statistically significant. These results suggest that inflammatory reaction is less intense in tissues categorized in Group 2.

The present results suggest that a chronic bacterial challenge from infected root canal causes the expression of two important cytokines, TNF-alpha and IL-6, which play a key role in periapical pathogenesis as potent bone resorption-stimulating mediators.
